# Put your money where your merch is? Disadvantaged group members' evaluations of symbolic corporate allyship with and without a material component

**DOI:** 10.1111/bjso.70149

**Published:** 2026-10-25

**Authors:** Lea Hartwich, Julia C. Becker

**Affiliations:** ^1^ Department of Psychology Osnabruck University Osnabruck Germany

**Keywords:** allyship, corporate allyship, performative allyship, social justice, social movements

## Abstract

As it has become increasingly common for companies to claim affinity with social movements, backlash against performative instances of corporate allyship has also been on the rise. Across three preregistered experimental studies in the contexts of Pride Month, Black History Month, and International Women's Day (total *N* = 659), we investigate marginalized groups members' perceptions of corporate allyship. We find that when support is not merely symbolic but includes material contributions in the form of donations to community organizations, it is perceived as less motivated by self‐interest (Studies 1 and 3), and more motivated by outgroup concerns (all studies) and moral values (Study 1). It is also considered more authentic (Studies 1 and 3), legitimate (Studies 1 and 3), effective (Study 1) and desirable (Study 1). Internal meta‐analyses show robust support for the effects on self‐focused motives, outgroup‐focused motives, authenticity, effectiveness and legitimacy, but only tentative support for the effects on moral motives and desirability. We discuss these results in relation to the broader literature on allyship and its heterogenous motivations and impacts, as well as the current political climate where there is a trend of companies stepping back from allyship under more conservative governments.

## INTRODUCTION

From Starbucks' support for equal marriage (Dodd & Supa, 2014) to Super Bowl advertisements displaying messages of women's empowerment (Thompson, 2025) to Nike featuring Colin Kaepernick in its anniversary campaign (Kim et al., 2020), it has become more and more common practice in recent years for corporations to take public stances in support of marginalized social groups (Dodd & Supa, 2014; Kim et al., 2020). However, criticism of this practice is also becoming increasingly vocal. These complaints do not always come from those on the opposing side of the political spectrum, as they did for Gilette's commercial criticizing toxic masculinity in the wake of #metoo (Baggs, 2019). Marginalized groups themselves have also expressed concerns about corporations attempting to associate themselves with their causes in ways that are considered unhelpful or inappropriate. Be it ‘rainbow‐washing’ by companies manufacturing their Pride merchandise in countries that criminalize queerness (Barker, 2022) or Walmart's culturally ‘tone‐deaf’ Juneteenth products advertised by White models (Meade, 2022; Neal, 2022), backlash frequently follows in the wake of such campaigns. Previous studies have shown that companies' attempts to take a stance on social issues are particularly badly received when they are seen as performative (Ponce de Leon et al., 2024; Skarmeas & Leonidou, 2013; Spielmann et al., 2023; Yim, 2021). In this research, we were interested in how marginalized group members make these judgements. Specifically, we conducted three experimental studies in the contexts of corporate allyship around Pride, Black History Month and International Women's Day to investigate how material, compared to purely symbolic, support affects whether allyship is seen as performative (i.e., motivated by self‐interest rather than moral values or solidarity, inauthentic and ineffective) and evaluated as less legitimate and desirable.

### What is corporate allyship?

The term corporate allyship includes ‘practices that companies, brands and other organizational entities engage in to support and show solidarity with a marginalized group or social movement’ (Thai & Nylund, 2024, p. 132). This is closely related to Corporate Social Advocacy (CSA), a concept referring to companies taking a public stance on controversial or contentious political issues (Dodd & Supa, 2014). It differs from Corporate Social Responsibility (CSR), which describes corporations' efforts to fulfil, or be seen as fulfilling, widely accepted ethical, social and environmental responsibilities that are often directly related to their business operations (Dodd & Supa, 2014; Nalick et al., 2016). Thus, unlike CSR, corporate allyship or CSA contains the risk of alienating some stakeholders by supporting others (Dodd & Supa, 2014). Research has shown that through these kinds of activities, companies can win over customers with congruent beliefs but risk the loss of those who do not agree with their position (Abitbol et al., 2018; Dodd & Supa, 2015).

However, it is not only those on the opposing side of a contested issue who may take a negative view of a corporation weighing in on social or political topics. Far from every CSA or allyship campaign is well‐received by those it is intended to appeal to, and it is not rare for companies to face criticism in the wake of such attempts (Abitbol et al., 2018; Baggs, 2019; Ponce de Leon et al., 2024). These critiques often revolve around perceptions of performativity, meaning that a corporation's actions are seen as self‐serving, disingenuous, low‐impact, or all of the above (Kutlaca & Radke, 2023; Ponce de Leon et al., 2024; Spielmann et al., 2023). Terms like wokewashing, pinkwashing, greenwashing or rainbow‐washing have been coined to describe hypocritical conduct by corporations or attempts to use allyship and social responsibility mainly in an attempt to improve the company's image or increase its bottom line (Austin et al., 2019; Rudnicka & Reichel, 2024).

Corporate allyship can take many forms, both internal, such as Diversity, Equity and Inclusion (DEI) initiatives in the workplace, and external, such as public statements or social media campaigns (Stephenson, 2024). Here, we focus on one external strategy that is both common and frequently draws criticism, namely, the practice of corporations adopting a marginalized community's symbols or aesthetics for their products. While this can increase visibility, it often does not offer community members any other benefits, apart from the opportunity to buy those products, usually at a premium (Barker, 2022). It has been argued that this can seem like a money grab (Barker, 2022) and make marginalized people feel commodified (Ponce de Leon et al., 2024). A particular point of criticism has been that usually none or very little of the money generated from this type of merchandise flows back to community organizations in the form of donations (Barker, 2022).

In this research, we investigate whether marginalized group members perceive this type of corporate allyship differently if that criticism is addressed, that is, if support is not merely symbolic, but also material, in the form of proceeds from the products going to community organizations. We expect that, compared to merely symbolic support, the addition of material support should reduce perceptions of performativity and increase positive evaluations of allyship.

### Performativity and allyship evaluations

Performativity is a term frequently used in critical evaluations of (corporate) allyship, both colloquially and in the marketing and public relations literature, which often focuses on how corporations can avoid perceptions of performativity in their campaigns. It is sometimes used interchangeably with inauthenticity or self‐interest (Ponce de Leon et al., 2024; Spielmann et al., 2023; Xue & Mattila, 2024), which in turn are also not always clearly delineated from each other. For example, genuine versus self‐serving motives are considered a facet of allyship authenticity in some definitions, but not others (Alhouti et al., 2016; Joo et al., 2019; Mirzaei et al., 2022).

For the purposes of this research, we draw on a definition of performative allyship from the social psychological literature proposed by Kutlaca and Radke (2023), which integrates several dimensions of allyship evaluations. They argue that performative actions are those that stem from self‐serving motives (or personal motivation, in the case of individual allies), meaning they are primarily concerned with accruing benefits for the allies themselves, in contrast to genuine motives that stem from moral commitments (morality motivation) and solidarity with marginalized groups (outgroup‐focused motivation), which prioritize social change (Radke et al., 2020). They further define performative actions as not requiring sacrifices from allies and contributing little to social change or challenging the status quo (Kutlaca & Radke, 2023). Building on this understanding of performative allyship, we investigate whether adding a material component to symbolic actions affects perceptions of performativity on three dimensions: motives, authenticity and effectiveness.

Research has shown that perceived motives affect reactions to corporate allyship. If a company's allyship is seen as motivated by self‐interest, such as considerations about its reputation or bottom line, it is likely to see a decline in consumers' trust and buying intentions (Mohr et al., 2001; Skarmeas & Leonidou, 2013; Theriault, 2020; Yoon et al., 2006; Zasuwa, 2017). Allyship that is seen as value‐driven, on the other hand, tends to have the opposite effect (Mohr et al., 2001; Skarmeas & Leonidou, 2013; Yim, 2021). These consequences on performance explain why companies are keen to avoid appearing self‐interested.

Authenticity is a related and overlapping concept, which captures to what extent a corporation's allyship is seen as genuine, committed and congruent with its other activities (Ahmad et al., 2024; Alhouti et al., 2016; Joo et al., 2019; Lim & Young, 2021). In an era where brands are tied to consumers' identities and people choose products not only based on practical or economic considerations but also based on the image and values they hope to express through their choice, being seen as authentic has become an important goal for corporations (Escalas, 2004). As with self‐interested motives, perceptions of inauthenticity are a central reason why corporate allyship campaigns receive backlash (Joo et al., 2019; Lim & Young, 2021; Xue & Mattila, 2024; Yim, 2021).

It has been proposed that corporate allyship should be perceived as higher in authenticity and lower in self‐interest when it is associated with costs to the ally (Mirzaei et al., 2022). This may be explained by Kelley's model of causal attribution, which proposes that actions incurring costs or risks are more likely to be attributed to intrinsic motivations (Kelley, 1973). Indeed, recent research based on this model has shown that advantaged group allies were seen as more sincere when they supported equality in the presence of inhibitory causes (Moser & Wiley, 2025). Given that material, compared to symbolic, allyship involves more costs for the company, it should thus be seen as more authentic, more strongly motivated by moral and outgroup concerns, and less motivated by self‐interest.

Finally, allyship performativity is evaluated based on its effectiveness, meaning the extent to which it actually benefits the target community and contributes to social change. Allyship is seen as effective when it is characterized by an alignment between the allies' actions and the needs and expectations of the marginalized group (Kutlaca & Radke, 2023; Pietri et al., 2024) and respects the autonomy of that group (Collier‐Spruel & Ryan, 2024). Compared to symbolic actions, material support should be seen as more effective since it better fulfils these criteria. It puts resources directly into the hands of the community, which can put them to use to improve the situation of its members and do so in the ways that it deems best.

In addition to these facets of performativity, we also investigate two further evaluative dimensions of corporative allyship. The first is legitimacy, meaning whether marginalized group members consider it appropriate for a corporation to associate itself with the community, use its symbols, participate in events, and present itself as an ally. This is particularly relevant, because the experience of seeing companies using such symbols or declaring themselves aligned with the community in ways that are felt to be inappropriate and unwelcome risks being particularly disempowering. The second is general positive evaluation or desirability, that is, whether marginalized group members approve of the company and its allyship and would like to see more companies engaging in similar types of actions. These evaluations have been shown to be more positive when a company's allyship entails costs rather than rewards (Thai & Nylund, 2024) and we would expect a similar pattern for material‐plus‐symbolic versus symbolic‐only allyship.

### The present research

When allyship is perceived as performative, this can have negative effects on corporations in the form of reputational damage or financial performance (Dodd & Supa, 2014, 2015; Will & Carriere, 2023). More importantly, while the effects on marginalized group members have not been the primary focus of study, it stands to reason that seeing their identities commodified, that is, used to sell products without deeper engagement or substantial support, is an alienating experience (Barker, 2022; Neal, 2022). From research on allyship outside of the corporate context, we know that actions motivated by self‐serving goals can not only backfire but can have detrimental effects on marginalized group members and movements (Droogendyk et al., 2016; Estevan‐Reina et al., 2021; Hasan‐Aslih et al., 2019). Similarly, allyship that is seen as ineffective because it fails to advocate, empower and challenge bias is linked to reduced psychological safety and increased anxiety for marginalized group members (Collier‐Spruel & Ryan, 2024). Moreover, exposure to performative allyship can reduce marginalized group members' trust in allies more generally (Grapin & Goldie, 2023). Therefore, it is particularly important that corporations, which in many cases have extensive resources and influence at their disposal, engage in forms of allyship that are seen as genuine and desirable by the communities they target.

Our research aimed to contribute to this by testing the hypothesis that symbolic‐plus‐material, compared to symbolic‐only support, is received by marginalized group members as less performative and more desirable and legitimate. To our knowledge, there have been no prior studies investigating this distinction, though research has started to look at the effect of costs associated with corporate allyship. Ponce de Leon and colleagues showed that allyship statements released by corporations in the wake of George Floyd's murder were evaluated more positively by Black participants when they contained reputationally or resource‐costly elements (Ponce de Leon et al., 2024). Thai and Nylund found that LGBTQIA+ participants saw a company's allyship as more genuine and evaluated it more positively when it led the company to suffer a financial loss, as opposed to a gain or no change in profits (Thai & Nylund, 2024). However, in this case, cost was conceptualized as a loss or sacrifice on the part of the corporation, but not one that materially benefitted the community in question. This distinction is important because it is possible for allyship to have substantial negative outcomes for a company without necessarily providing any benefit to the marginalized group, just as the reverse may be the case. For example, if a company's donation is tax‐deductible or outweighed by additional profits, allyship can provide material benefits without automatically incurring substantial costs. Thus, we expand on previous research by looking at how the financial outcome for the community, rather than the corporation itself, affects evaluations of allyship.

To this end, we experimentally manipulated whether corporations engage in allyship that is merely symbolic or allyship that involves an additional material benefit, in the form of financial donations, to the group the actions are meant to support. We then investigated disadvantaged group members' evaluations of this allyship on five outcome dimensions, covering perceived motives, authenticity, effectiveness, legitimacy and desirability. We expect that material allyship will lead participants to infer less self‐interested motivation (H1), stronger moral motivation (H2) and stronger outgroup‐focused motivation (H3). We also expect that participants will perceive the corporations' allyship as more authentic (H4) and effective (H5) and evaluate it as more legitimate (H6) and desirable (H7). We conducted the same experiment across three different contexts; therefore, the methods and results are presented together below. Because not all results were consistent across studies, we also conducted an internal meta‐analysis for a more reliable estimate of effect sizes.

## STUDIES 1–3

All three studies followed the same procedure and employed the same measures and analyses. We only varied the context and target group, with Study 1 (preregistration: https://aspredicted.org/xd99qn.pdf) investigating corporate allyship with the queer community during Pride Month, Study 2 (preregistration: https://aspredicted.org/xe9cd4.pdf) focusing on allyship with the Black community during Black History Month, and Study 3 (preregistration: https://aspredicted.org/u74jh6.pdf) looking at the context of allyship with women during International Women's Day. Participants provided informed consent before participating in the study and were subsequently debriefed. All materials, data, and analysis scripts are available in the SOM at the OSF depository: https://osf.io/psre9.

### Method

#### Participants

For Studies 1 and 2, power analysis for an ANOVA design with two groups was set to detect a medium sized effect, *f* = .25, *α* = .05, 1 − *β* = .95, and indicated a required sample size of 210 per study. For Study 3, power analysis was originally conducted for a 2 × 2 design to explore whether there was a moderating effect of participant group membership on evaluations (see preregistration), with women and minority genders as the marginalized group, as well as men as the privileged group included in the study. This would have required a sample size of 327 which was not reached after exclusions for attention check failure only left a sample of 200. Furthermore, only 82 men (49 after excluding attention check failures) participated in the study, resulting in very unequal group sizes. Therefore, in line with the other studies, only marginalized group members are included in the analysis.^1^ Post‐hoc power analysis indicated that the remaining sample of 151 marginalized group participants still yielded power of 1 − *β* = .86 to detect a medium effect. Sample characteristics for each study are reported in Table 1.

**TABLE 1:** Sample characteristics.

	Study 1	Study 2	Study 3
Country	Germany	United States	Germany
Recruitment strategy	Convenience sample (recruited by research assistants and via social media)	Commercial (recruited via Prolific and Bovitz)	Convenience sample (recruited by research assistants and via social media)
Initial *N* (symbolic/material) (complete cases)	298 (139/159)	Total: 510 (278/232) Prolific: 308 (153/155) Bovitz: 202 (125/77)	265 (136/129)
Inclusion criteria	Self‐identification as queer/LGBTQI+	Self‐identification as Black	Self‐identification as women or minority gender
*N* after inclusion criteria	298 (139/159)	Pre‐screened by panel	196 (102/94)
Attention check 1	Topic of the newspaper article		
*N* after attention check 1	293 (139/154)	T: 362 (197/165) P: 220 (106/114) B: 142 (91/51)	195 (102/93)
Attention check 2	Products sold by the target company		
*N* after attention check 2	291 (138/153)	T: 353 (190/163) P: 219 (106/113) B: 134 (84/50)	195 (102/93)
Attention check 3	Allyship actions taken by the target company		
*N* after attention check 3	244 (127/117)	T: 272 (172/100) P: 152 (95/57) B: 120 (77/43)	154 (99/55)
Attention check 4	Select “completely agree” (Study 1 + 3)/“agree” (Study 2)		
Final *N* (symbolic/material)	243 (127/116)	T: 265 (166/99) P: 151 (94/57) B: 114 (72/42)	151 (98/53)
*M*_age_ (SD)	26.57 (7.94)	43.35 (15.89)	30.73 (12.86)
Gender			
% women	56	57	95
% men	16	43	–
% non‐binary/other	28	0	5
Political orientation (1 = *liberal* to 7 = *conservative*)	*M* = 2.01, SD = 0.77	*M* = 3.48, SD = 1.47	–
Regular collective action engagement (participated at least 5 times)	41%	11%	24%

#### Procedure

Participants read a short newspaper article (see Appendix S1 for an example), which briefly introduced the concept of corporate allyship for the respective community and then focused on one fictitious company. In Study 1, this was a soft drink manufacturer called RefreshCo, and in Studies 2 and 3 an activewear brand called Move‐U and ActiveStyle respectively. The article quoted the company's spokesperson who explained how the company would support the community during Pride, Black History Month, or International Women's Day. In the symbolic‐only condition, this involved the sale of special edition products bearing, for example, the rainbow flag. In the symbolic‐plus‐material condition, the company pledged to donate the proceeds from the sale of these products to organizations dedicated to advancing queer, racial or gender equality respectively.

This was followed by three attention check items asking about the topic of the article, the type of products sold by the company, and the allyship actions it was engaging in. Participants could look at the article again while answering these questions. We slightly deviated from our preregistrations here. For Studies 1 and 3, we originally planned to only exclude participants who failed more than one attention check. However, because most attention check failures concerned the item asking about the key aspect of the manipulation, that is, the actions taken by the company, we instead excluded participants for any attention check failures. In Study 2, we had preregistered that we would exclude participants for any attention check failures. We made one exception here because one distractor item on the question about the company's actions (‘featuring Black athletes on its website’) received false positive replies from 127 participants which suggested to us that it may have been poorly chosen. In general, more participants failed the question about the company's allyship actions in the symbolic‐plus‐material condition compared to the symbolic‐only condition, resulting in unequal cell sizes in Studies 2 and 3. This may be due to participants in that condition having to select two options, whereas those in the symbolic‐only condition only had to select the option for the symbolic action. We conducted our analyses without excluding attention check failures for comparison, but except for the effect on effectiveness in Study 3 which reached significance (compared to *p* = .063 in the final sample), this did not affect results (see Table S1).

#### Measures

Details of the outcome measures are reported in Table 2. All items were measured on scales from *1* (completely disagree) to *7* (completely agree). Wordings were adapted to refer to the relevant company name, occasion, and target community.^2^

**TABLE 2:** Measures and reliability.

Construct	# items	Sample item	Reliability		
			Study 1	Study 2	Study 3
Self‐interested motive (Hartwich & Becker, 2026)	3	*Company* supports the community in order to improve its own image	*α* = .80	*α* = .75	*α* = .85
Moral motive (Hartwich & Becker, 2026)	2	The company is standing up for its principles	*r* = .65	*r* = .75	*r* = .73
Outgroup‐focused motive (Hartwich & Becker, 2026)	2^a^	The company's engagement primarily serves the interests of the community	*r* = .53	*α* = .79	*r* = .58
Authenticity (Alhouti et al., 2016)	6^b^	I believe that the company's allyship for the community is genuine	*α* = .91	*α* = .93	*α* = .93
Effectiveness (Radke et al., 2022)	4	I think the company is a helpful ally in the struggle for *community's* rights	*α* = .86	*α* = .92	*α* = .87
Legitimacy	5^c^	I think it is legitimate for the company to sell products decorated with *symbol*	*α* = .82	*α* = .92	*α* = .78
Desirability (Thai & Nylund, 2024)	3	I would like to see more corporations like *company* supporting the community	*α* = .89	*α* = .96	*α* = .91

^a^ The measure of outgroup‐focused motive included 3 items in Study 2.

^b^ The measure of authenticity included a 7th item in Study 3.

^c^ The legitimacy measure was shortened to 4 items in Study 3.

### Results and discussion

Analyses were carried out in R Version 4.5.2 (R Core Team, 2025). Correlations tables for the entire samples and by condition are available in Table S4. ANOVA (Table 3) provided support for our hypotheses, though this was not consistent across studies for all outcomes. Corporations engaging in symbolic‐plus‐material, compared to symbolic‐only allyship, received lower attributions of self‐interest in Studies 1 and 3 (in line with H1). They were attributed more moral motives only in Study 1 (H2), but more outgroup‐focused motives across all studies (H3). In Studies 1 and 3, but not in Study 2, they were perceived as significantly more authentic (H4) and legitimate (H6). Ratings of effectiveness (H5) and desirability (H7) were higher only in Study 1.

**TABLE 3:** Cell means and standard deviations as a function of allyship type, with mean difference tests.

Outcome	Study 1						Study 2						Study 3					
	*M* (SD)		*F*(1, 241)	*p*	*η* ^2^	95% CI	*M* (SD)		*F*(1, 263)	*p*	*η* ^2^	95% CI	*M* (SD)		*F*(1, 149)	*p*	*η* ^2^	95% CI
	Sym. (127)	Mat. (116)					Sym. (166)	Mat. (99)					Sym. (98)	Mat. (53)				
Self‐focused motive	5.85 (0.95)	4.97 (1.03)	48.52	<.001	.17	0.09, 0.25	4.89 (1.39)	4.69 (1.42)	1.25	.265	.004	0.00, 0.03	5.54 (1.11)	4.29 (1.43)	35.30	<.001	.19	0.09, 0.30
Moral motive	3.91 (1.18)	4.47 (1.03)	15.36	<.001	.06	0.02, 0.13	5.21 (1.45)	5.34 (1.21)	0.59	.444	.002	0.00, 0.03	4.42 (1.15)	4.30 (1.24)	0.34	.564	.002	0.00, 0.04
Outgroup‐focused motive	2.68 (1.06)	3.94 (1.11)	81.66	<.001	.25	0.17, 0.34	4.56 (1.44)	4.92 (1.26)	4.21	.041	.02	0.00, 0.06	3.14 (1.26)	4.14 (1.41)	20.12	<.001	.12	0.04, 0.22
Authenticity	2.77 (1.08)	3.75 (1.10)	49.95	<.001	.17	0.09, 0.26	4.53 (1.55)	4.65 (1.45)	0.41	.520	.003	0.00, 0.02	3.07 (1.13)	3.67 (1.33)	8.68	.004	.06	0.01, 0.14
Effectiveness	3.41 (1.16)	3.76 (1.22)	5.50	.020	.02	0.00, 0.07	5.07 (1.48)	5.22 (1.34)	0.69	.406	.003	0.00, 0.03	3.61 (1.10)	3.98 (1.29)	3.51	.063	.02	0.00, 0.09
Legitimacy	3.79 (1.12)	4.25 (1.19)	9.33	.003	.04	0.00, 0.10	5.11 (1.39)	5.29 (1.27)	0.73	.393	.003	0.00, 0.03	4.52 (1.02)	5.01 (1.22)	6.96	.009	.04	0.00, 0.13
Desirability	4.27 (1.24)	4.83 (1.35)	11.25	<.001	.04	0.01, 0.11	5.65 (1.48)	5.68 (1.27)	0.03	.854	.0001	0.00, 0.01	5.01 (1.26)	5.20 (1.27)	0.82	.367	.01	0.00, 0.05

A noticeable pattern in these results is the differences between Study 2 and the others. This manifests in two ways. For one, judgements of corporate allyship on the whole appear to be more favourable in Study 2, compared to Studies 1 and 3. At the same time, although the mean differences point in the expected direction, only one of them reached significance. It is possible that the effect of symbolic‐only versus symbolic‐plus‐material allyship does not apply to the context of racial justice, or the national context of the United States, to the same extent that it does to the contexts of gender and sexuality, or the national context of Germany. However, considering that many well‐known examples of backlashes against symbolic corporate allyship campaigns considered performative come from precisely that context (Abitbol et al., 2018; Neal, 2022), this does not seem the most likely explanation. Another possible reason for the more uniformly positive perceptions of allyship would be that it is considered particularly high‐risk in this context and thus seen as potentially costly and therefore more genuine (Kelley, 1973; Moser & Wiley, 2025), regardless of its nature. However, a measure of risk included in Study 2 indicated that participants in both conditions thought that Black and White people would likely view the campaign positively and that the company was unlikely to lose business or alienate customers (see Table S1). As this measure was not included in the other studies, we cannot draw comparisons in this case.

There are also a number of explanations related to sample characteristics. For one, the Study 2 sample was older on average than the others and a measure of political orientation included in Studies 1 and 2 revealed that it was also more conservative. Moreover, almost half of the participants in Study 1 and a quarter in Study 3 indicated that they had participated in some form of collective action on their community's behalf more than five times in the past, compared to only 11% in Study 2 (where 42% had never done so). Taken together with the generally less favourable perceptions of corporate allies regardless of condition in Studies 1 and 3, it is possible that younger, more progressive and activist members of marginalized groups are more critical and discerning in their judgements and have a higher bar of what is considered good allyship. We did preregister explorative analyses testing for moderating effects of political orientation and past collective action participation, and we additionally tested for a moderating effect of age. There were no interactions between condition and political orientation. Only one interaction with past collective action reached significance, which was for outgroup‐focused motive in Study 1, where past collective action participation had a negative effect in the symbolic‐only but not the symbolic‐plus‐material allyship condition, suggesting that more activist participants were more critical of corporations engaging in allyship without a material element. For age, we found a few significant interactions with condition, which were somewhat inconsistent. There were interactions for self‐focused motive in Studies 1 and 3. In Study 1, there was a negative effect in the symbolic‐only condition, meaning that older participants suspected less self‐focused motivations in corporations engaging in symbolic allyship. On the other hand, in Study 3, there was a positive effect in the symbolic‐plus‐material condition, suggesting that older participants were more suspicious of corporations also making financial donations. Additionally, in Study 3, there was an interaction for moral motive, with a negative effect of age only in the symbolic‐plus material condition, suggesting that older participants thought these companies less likely to be acting on their moral principles (see Table S2).

## INTERNAL META‐ANALYSIS

In cases like ours, where a series of conceptually comparable studies produce the same directional trend but not all yield significant effects, it has been recommended to conduct an internal meta‐analysis to get a better estimate of effect sizes (Goh et al., 2016; Maner, 2014). We used the *metafor* (Viechtbauer, 2010) package to conduct fixed effects meta‐analyses. This choice was determined by the number of studies, as random effects models are not recommended when there are fewer than five (Andrade, 2025). Fixed effects models are suitable in cases where the procedure is similar across studies, which is the case for our experiments with regard to materials and measures, although their different contexts and recruitment methods constitute a limitation. The results show significant effects for all outcome variables (Table 4). These ranged from small Cohen's *d* for moral motives, effectiveness, legitimacy and desirability to medium effects for self‐centered motives, outgroup‐focused motives and authenticity.

**TABLE 4:** Results of internal meta‐analysis.

	*M*_d_	SE	*Z*	*p*	95% CI	
					Lower	Upper
Self‐focused motive	−0.60	0.08	−7.31	<.001	−0.76	−0.44
Morality motive	0.21	0.08	2.57	.010	0.05	0.36
Outgroup‐focused motive	0.69	0.08	8.34	<.001	0.53	0.85
Authenticity	0.47	0.08	5.78	<.001	0.31	0.63
Effectiveness	0.22	0.08	2.79	.005	0.07	0.38
Legitimacy	0.30	0.08	3.76	<.001	0.15	0.46
Desirability	0.21	0.08	2.57	.010	0.05	0.36

For comparison, random effects models, heterogeneity estimates and leave‐one‐out sensitivity analyses are reported in Table S5. With random effects, moral motives and desirability no longer differ significantly between conditions. Tests of heterogeneity were significant for self‐focused motive, moral motive, outgroup‐focused motive and authenticity. The effects on self‐focused motives and authenticity do not reach significance if Studies 1 or 3 are left out. The effect of outgroup‐focused motive does not reach significance if Study 3 is left out and only just becomes significant if Study 1 is left out. The results also show that, with the exception of moral motives, all effects become significant and tests of heterogeneity insignificant if Study 2 is left out.

## GENERAL DISCUSSION

Our studies provided at least some support for each hypothesis about the differences in perceived performativity and evaluation between symbolic‐only and symbolic‐plus‐material corporate allyship. We found that when corporations did not merely sell special edition products purporting to celebrate a community but donated the proceeds to community organizations, their allyship was seen as less self‐interested (Studies 1 and 3), more motivated by moral values (Study 1) and solidarity with the group (all studies), as well as more authentic (Studies 1 and 3), effective (Study 1), legitimate (Studies 1 and 3) and desirable (Study 1). While results were largely consistent between Studies 1 and 3, only one effect reached significance in Study 2. However, the means did show a tendency in the expected direction even in Study 2, and the internal meta‐analysis also supports the idea that there are small to medium‐sized effects of action type on the perception and evaluation of corporations engaging in allyship. It is important to note that meta‐analyses no longer showed significant results for moral motives and desirability if random rather than fixed effects models were used, meaning that these results should be interpreted with caution.

### Theoretical and practical implications

Our findings are in line with previous studies showing that companies are evaluated more positively when their allyship involves costs rather than rewards (Ponce de Leon et al., 2024; Thai & Nylund, 2024). They also fit with Kutlaca and Radke's theorizing about the nature of performative allyship, showing that more substantial allyship is perceived differently by recipients both in terms of motivations and in terms of impact (Kutlaca & Radke, 2023). It expands on previous research on CSR, CSA and corporate allyship, which have often focused on customer reactions or the outcomes for companies' market value and sales (Coetzee et al., 2023; Dodd & Supa, 2014, 2015; Mohr et al., 2001; Skarmeas & Leonidou, 2013) by centring the reactions of marginalized group members and empirically showing that they do not consider every type of corporate allyship to stem from genuine solidarity or view it as particularly helpful. It also adds to previous research demonstrating more positive perceptions of corporate allyship based on costs versus benefits to the company (Thai & Nylund, 2024) by investigating the new dimension of material benefits to the community.

In Studies 1 and 3 in particular, company evaluations on many of the dimensions we investigated were relatively negative, even in the material allyship condition. Most notably, perceptions of authenticity consistently fell below the neutral scale midpoint, whereas self‐focused motives lay above it. Effectiveness, too, was rated as neutral or below, meaning that on the whole, many participants considered corporate allyship not to be genuine, nor very likely to contribute to social change. This indicates that both forms of allyship were seen as performative to some extent. It is worth noting that while financial donations can make a material difference to communities, they do not necessarily come at a substantial cost to companies. Thus, if, based on attribution theory, perceptions of genuine allyship are at least partly based on cost or sacrifice (Kelley, 1973), it is possible that marginalized group members did not consider these donations to constitute a significant enough sacrifice to signal authenticity. It is also possible that the combination of symbolic and material support was still regarded with some scepticism because of a general wariness of the symbolic element. Perhaps disadvantaged group members have grown cynical about corporations using their symbols or aesthetics in the hopes of increasing product sales to the point where even additional financial contributions do not entirely remove perceptions of inauthenticity and self‐interest. This is understandable, given that these donations are often tax‐deductible and have a very small impact on the bottom lines of major corporations. It may well make economic sense for a company to incur such relatively minor costs for the benefit of improving its image and winning over new customers. If corporations genuinely wish to win the trust of disadvantaged groups and become more welcome allies, they may consider building more substantive ties with communities and charities and engaging in consistent and sustained allyship (Ciordas et al., 2026) even under adverse circumstances (Moser & Wiley, 2025).

Moving beyond the findings of these studies, there are good reasons for disadvantaged group members to regard even less performative corporate allyship with a grain of scepticism. For one, it has been argued that the co‐option of social movements by companies leads to more radical demands for social change being sacrificed for palpability and mainstream appeal (Tatchell, 2019). This seems almost inevitable because it would make little sense for corporations to support social movements that do not already have broad appeal and mainstream support, given that they are necessarily subject to the pressures of a competitive marketplace. Companies are constantly engaged in Strategic Issue Management, meaning they seek to align their organizational behaviour with societal and stakeholder expectations about the ways in which organizations should behave (Dodd & Supa, 2014). Marginalized groups are only one such stakeholder group and, except perhaps in the case of a company operating in a niche market where a specific marginalized group constitutes its primary customer base, their influence is necessarily limited compared to that of general public opinion or more powerful societal actors. Appeals for corporate partners willing to challenge the systems they profit from and to commit to structural change (Awori, 2025) ignore these systemic pressures and may raise expectations of or reliance on companies as agents for social change that can become a problem for social movements, especially when societal climates turn more hostile towards them.

Recent developments in the United States are a powerful example of this. Many corporations have scrapped or scaled down their DEI initiatives and similar efforts under pressure from conservative interest groups and the new presidential administration, openly citing concerns about the political landscape, federal contracts and hits to their bottom‐lines as reasons (Meyersohn & McKend, 2025; The Guardian, 2025). Many major brands have also reduced their Pride campaigns in recent years, and this has not only affected the availability of Pride‐themed products, but also corporate contributions to LGBTQIA+ charities and NGOs (Czachor, 2024). Even though there may be the occasional outlier, corporations generally have no incentive to support marginalized groups in adverse climates and their quick turn towards DEI during the height of the Black Lives Matter protests in 2020 as well as their sharp turn away from it a mere 4 years later illustrates this point.

### Limitations and directions for future research

While our internal meta‐analysis provides support for all hypotheses tested in this research, the fact remains that there were differences across the three studies, most notably with Study 2. We did not have access to representative samples that would have kept other characteristics, such as national context, age, or political orientation the same across studies, which means that we cannot definitively say which, if any, of those factors may have contributed to the differences in results. Given the high number of attention check failures, we also cannot rule out that inattentive responding had something to do with it. Future research may be able to more systematically investigate the effects of merely symbolic versus material allyship across different contexts.

It is also important to note that even within the same context, marginalized groups are not monoliths and thus their attitudes towards social movements and their corporate allies are bound to be heterogeneous. We investigated some of that variation by testing for the moderating roles of age, identity centrality, political orientation, activism and overall attitudes towards Corporate Social Advocacy. These analyses did not yield any consistent results across studies, which may be due to samples being relatively small and homogeneous. However, some interaction effects did emerge for activism and age. It would be of interest to look more closely at how different agendas and priorities affect attitudes towards corporations trying to engage with social movements. For example, marginalized group members who believe that their group's goal should be to achieve substantial systemic changes rather than representation or equality within existing systems should be more likely to reject corporate allyship regardless of the form it takes. On the other hand, more market‐oriented individuals may actually prefer to be treated as customers and view donations to the community as disempowering charity.

Regarding the characteristics of the allyship itself, our research compared two scenarios of a corporation selling special edition products referencing the disadvantaged group's struggle for equality. In one condition, this was the whole extent of the allyship, whereas in the other, a material benefit to the community in the form of donations of profits was added. A third possibility that we did not investigate would be for a corporation to donate without engaging in symbolic allyship. On the one hand, such allyship may be received more positively than the combination of material and symbolic support because it is seen as less self‐interested. On the other hand, depending on the context, it might also give the impression that the company is trying to keep its support less visible in order to avoid losing customers who are opposed to progressive causes, which would constitute an avoidance of allyship costs (Moser & Wiley, 2025; Thai & Nylund, 2024). It would be an interesting avenue for future research to shed light on the conditions under which allyship without the symbolic element is evaluated more or less positively.

Moreover, our studies focused on a one‐off, event‐related instance of support. Previous research focusing on individual allies has shown that perceptions of internal motivation and trustworthiness are shaped by allyship consistency (Ciordas et al., 2026). It stands to reason that the same applies to corporate allyship and that consistency especially affects evaluations of symbolic actions, which are likely to be more welcome from corporations who consistently support the disadvantaged group and are thus seen as more entitled to use its symbols on their products.

Finally, research on collective action and allyship has frequently been criticized for being disproportionately focused on contexts where repression is relatively low (Vollhardt & Bou Zeineddine, 2024). However, corporate allyship, as pointed out above, is not a phenomenon that is often encountered in repressive contexts. Usually, corporations bend to discriminatory laws and norms, such as movie studios removing scenes with queer content for distribution in countries where so‐called ‘homosexual propaganda’ is outlawed, or even pre‐emptively tailoring their content to appease censors (BBC News, 2020). One opportunity to study a relatively more repressive context may be the current “war on DEI” in the United States. Given that our US data was collected at the end of 2024, before the start of the second Trump administration, it would be of interest to investigate whether the results look different in the current climate.

## CONCLUSION

Across three studies in the contexts of Pride, Black History Month, and International Women's Day, we investigated how the symbolic‐only versus symbolic‐plus‐material nature of corporate allyship affects disadvantaged group members' perceptions and evaluations of companies. Pooled results suggest that compared to merely symbolic support, financial donations to community organizations were seen as less performative and judged to be more legitimate and desirable, although discrepancies between random and fixed effects models suggest that the effects on moral motives and desirability may need to be interpreted cautiously. There were also some inconsistencies across studies, with only Study 1 (Pride) showing significant effects on all outcomes. In Study 2 (Black History Month), only the effect on perceived outgroup motivation was significant. In Study 3 (International Women's Day), there were significant effects on all outcomes except for moral motivation, effectiveness, and desirability. In two of our samples, ratings indicated a general scepticism towards corporate allies regardless of their specific actions, which may result from an awareness of the structural limitations applying to this type of allyship.

## AUTHOR CONTRIBUTIONS

**Lea Hartwich:** Conceptualization; methodology; investigation; formal analysis; writing – original draft. **Julia C. Becker:** Writing – review and editing; conceptualization.

## ACKNOWLEDGEMENTS

Open Access funding enabled and organized by Projekt DEAL.

## FUNDING INFORMATION

No funding was received for this research.

## CONFLICT OF INTEREST STATEMENT

The authors have no conflicts of interest to declare.

## ETHICS STATEMENT

Ethics approval was obtained from the ethics committee at the authors' university. All participants provided informed consent to participate in the studies and were debriefed afterwards.

## Supporting information


Table S1

Table S2

Table S3

Table S4

Table S5

Appendix S1

## Data Availability

All materials, data and analysis scripts are available at the OSF depository: https://osf.io/psre9.

## Footnotes

^1^
We conducted the originally planned 2 × 2 ANOVA for group × condition and found neither main effects of group membership nor interaction effects, but because of the small sample and unequal cell sizes, these results should be interpreted with caution (see Table S2).

^2^
In Studies 1 and 2, we also measured ingroup identification and general corporate allyship attitudes before the manipulation. There were no significant interaction effects with condition (see Table S2). The following additional outcome measures were included: collective action intentions (Studies 1 and 2), consumer engagement (Study 1), self‐brand connection (Study 1), employer branding (Study 3), perceived allyship risk (Study 3). Only consumer engagement was significantly higher in the symbolic‐plus‐material condition (see Table S3). Items can be found in the respective study materials.
