# Supplementary material for: Put your money where your merch is? Disadvantaged group members' evaluations of symbolic corporate allyship with and without a material component
Source: Br J Soc Psychol. 2026 Oct 25;65(4):e70149. doi: 10.1111/bjso.70149 (PMC13696842; doi:10.1111/bjso.70149)
Supplement: Supplementary file 1 [file BJSO-65-0-s001.docx]

SOM Table E

*Results of random effects meta-analyses with estimated heterogeneity and leave-one-out sensitivity analyses.*

|  | *M*_d_ | *SE* | *Z* | *p* | 95% CI | | Tau^2 | *SE* | Q (2) | *p* |
| --- | --- | --- | --- | --- | --- | --- | --- | --- | --- | --- |
|  |  |  |  |  | lower | upper |  |  |  |  |
| **Self-focused motive** | **-.67** | **.27** | **-2.46** | **.014** | **-1.21** | **-0.14** | **.20** | **.22** | **22.69** | **<.001** |
| - Study 1 | -.57 | .43 | -1.31 | .191 | -1.42 | 0.28 | .35 |  | 15.49 | <.001 |
| - Study 2 | -.93 | .11 | -8.64 | <.001 | -1.14 | -0.72 | .00 |  | 0.30 | .584 |
| - Study 3 | -.51 | .37 | -1.38 | .168 | -1.24 | 0.22 | .26 |  | 16.18 | <.001 |
| Moral motive | .18 | .18 | 1.00 | .318 | -0.17 | 0.52 | .07 | .09 | 9.17 | .010 |
| - Study 1 | .03 | .10 | 0.25 | .806 | -0.17 | 0.22 | .00 |  | 0.85 | .357 |
| - Study 2 | .21 | .30 | 0.70 | .485 | -0.38 | 0.80 | .16 |  | 7.90 | .005 |
| - Study 3 | .30 | .20 | 1.46 | .144 | -0.10 | 0.70 | .07 |  | 5.01 | .025 |
| **Outgroup-focused motive** | **.72** | **.27** | **2.73** | **.006** | **0.20** | **1.24** | **.19** | **.21** | **22.87** | **<.001** |
| - Study 1 | .49 | .25 | 2.00 | .046 | 0.09 | 0.98 | .10 |  | 5.21 | .022 |
| - Study 2 | .97 | .20 | 4.86 | <.001 | 0.58 | 1.36 | .06 |  | 3.21 | .073 |
| - Study 3 | .70 | .45 | 1.58 | .115 | -0.17 | 1.59 | .39 |  | 22.69 | <.001 |
| **Authenticity** | **.48** | **.24** | **2.02** | **.043** | **0.02** | **0.96** | **.15** | **.18** | **19.52** | **<.001** |
| - Study 1 | .27 | .21 | 1.30 | .192 | -0.14 | 0.68 | .06 |  | 3.78 | .052 |
| - Study 2 | .71 | .20 | 3.56 | <.001 | 0.32 | 1.10 | .06 |  | 3.34 | .068 |
| - Study 3 | .49 | .41 | 1.90 | .234 | -0.31 | 1.29 | .32 |  | 19.50 | <.001 |
| **Effectiveness** | **.22** | **.08** | **2.79** | **.005** | **0.07** | **0.38** | **.00** | **.02** | **1.45** | **.484** |
| - Study 1 | .18 | .10 | 1.75 | .079 | -0.02 | 0.38 | .00 |  | 0.97 | .325 |
| - Study 2 | .30 | .10 | 2.92 | .004 | 0.10 | 0.50 | .00 |  | 0.01 | .921 |
| - Study 3 | .20 | .09 | 2.09 | .036 | 0.01 | 0.38 | .00 |  | 1.09 | .297 |
| **Legitimacy** | **.31** | **.10** | **3.07** | **.002** | **0.11** | **0.51** | **.01** | **.03** | **3.00** | **.224** |
| - Study 1 | .27 | .15 | 1.73 | .083 | -0.04 | 0.57 | .03 |  | 2.13 | .145 |
| - Study 2 | .41 | .10 | 4.00 | <.001 | 0.21 | 0.62 | .00 |  | 0.05 | .823 |
| - Study 3 | .26 | .13 | 2.00 | .045 | 0.01 | 0.52 | .02 |  | 2.12 | .146 |
| Desirability | .20 | .13 | 1.57 | .117 | -0.05 | 0.46 | .03 | .05 | 5.24 | .073 |
| - Study 1 | .07 | .10 | 0.66 | .511 | -0.13 | 0.27 | .00 |  | 0.36 | .546 |
| - Study 2 | .31 | .14 | 2.24 | .025 | 0.04 | 0.59 | .02 |  | 1.73 | .189 |
| - Study 3 | .23 | .21 | 1.10 | .272 | -0.18 | 0.63 | .07 |  | 5.10 | .024 |
