# Supplementary material for: Put your money where your merch is? Disadvantaged group members' evaluations of symbolic corporate allyship with and without a material component
Source: Br J Soc Psychol. 2026 Oct 25;65(4):e70149. doi: 10.1111/bjso.70149 (PMC13696842; doi:10.1111/bjso.70149)
Supplement: Supplementary file 2 [file BJSO-65-0-s003.docx]

SOM Table A

*Cell means and standard deviations as a function of allyship type, with mean difference tests.*

| Outcome | *M (SD)* | |  |  |  |
| --- | --- | --- | --- | --- | --- |
|  | sym. | mat. | *F* | *df* | *p* |
| Collective action (Study 1) | 3.22  (1.13) | 3.22  (1.27) | 0.002 | 1, 241 | .968 |
| **Consumer engagement (Study 1)** | **2.46**  **(0.99)** | **2.80**  **(1.06)** | **6.40** | **1, 240** | **0.12** |
| Self-brand connection (Study 1) | 1.98  (1.01) | 2.17  (1.06) | 2.02 | 1, 239 | .156 |
| Collective action (Study 2) | 5.29  (1.51) | 5.00  (1.48) | 2.37 | 1, 263 | .125 |
| Risk 1 (r): Black people would approve (Study 2) | 5.42  (1.49) | 5.34  (1.49) | 0.15 | 1, 263 | .702 |
| Risk 2 (r): White people would approve (Study 2) | 4.37  (1.63) | 4.27  (1.61) | 0.21 | 1, 263 | .646 |
| Risk 3: Losing customers (Study 2) | 2.99  (1.69) | 3.33  (1.61) | 2.69 | 1, 263 | .102 |
| Risk 4: Alienating people (Study 2) | 3.13  (1.74) | 3.11  (1.69) | 0.01 | 1, 263 | .922 |
| Employer branding (Study 3) | 3.76 (1.28) | 4.18  (1.40) | 3.49 | 1, 148 | .064 |
