# Supplementary material for: Put your money where your merch is? Disadvantaged group members' evaluations of symbolic corporate allyship with and without a material component
Source: Br J Soc Psychol. 2026 Oct 25;65(4):e70149. doi: 10.1111/bjso.70149 (PMC13696842; doi:10.1111/bjso.70149)
Supplement: Supplementary file 3 [file BJSO-65-0-s004.docx]

Appendix I

Experimental Manipulation (Study 2) – material condition

Experimental Manipulation (Study 2) – symbolic condition
