# Supplementary material for: Put your money where your merch is? Disadvantaged group members' evaluations of symbolic corporate allyship with and without a material component
Source: Br J Soc Psychol. 2026 Oct 25;65(4):e70149. doi: 10.1111/bjso.70149 (PMC13696842; doi:10.1111/bjso.70149)
Supplement: Supplementary file 4 [file BJSO-65-0-s005.docx]

SOM Table B: Moderation analyses

Past collective action x condition

| Outcome | *F* | *df* | *p* |
| --- | --- | --- | --- |
| Self-focused motive |  |  |  |
| Study 1 | 0.79 | 1, 239 | .376 |
| Study 2 | 0.003 | 1, 261 | .954 |
| Study 3 | 1.58 | 1, 147 | .284 |
| Moral motive |  |  |  |
| Study 1 | 0.76 | 1, 239 | .382 |
| Study 2 | 0.00 | 1, 261 | .998 |
| Study 3 | 0.20 | 1, 147 | .658 |
| Outgroup-focused motive |  |  |  |
| **Study 1** | **6.07** | **1, 239** | **.014** |
| **Symbolic-only condition** | **b = -.26, SE = .08, 95% CI [-.41; -.10]** | | |
| Symbolic-plus-material condition | b = -.01, SE = .08, 95% CI [-.14; 16] | | |
| Study 2 | 1.43 | 1, 261 | .233 |
| Study 3 | 0.25 | 1, 147 | .560 |
| Authenticity |  |  |  |
| Study 1 | 2.63 | 1, 239 | .107 |
| Study 2 | 1.98 | 1, 261 | .161 |
| Study 3 | 1.71 | 1, 147 | .194 |
| Effectiveness |  |  |  |
| Study 1 | 1.35 | 1, 239 | .247 |
| Study 2 | 0.34 | 1, 261 | .562 |
| Study 3 | 1.66 | 1, 147 | .200 |
| Legitimacy |  |  |  |
| Study 1 | 1.49 | 1, 239 | .224 |
| Study 2 | 3.35 | 1, 261 | .068 |
| Study 3 | 0.23 | 1, 147 | .631 |
| Desirability |  |  |  |
| Study 1 | 1.19 | 1, 239 | .276 |
| Study 2 | 0.63 | 1, 261 | .427 |
| Study 3 | 0.21 | 1, 147 | .645 |

Age x condition

| Outcome | *F* | *df* | *p* |
| --- | --- | --- | --- |
| Self-focused motive |  |  |  |
| **Study 1** | **9.92** | **1, 238** | **.002** |
| **Symbolic-only condition** | **b = -.04, SE = .01, 95% CI [-.06; -.01]** | | |
| Symbolic-plus-material condition | b = .01, SE = .01, 95% CI [-.01; 03] | | |
| Study 2 | 0.12 | 1, 261 | .722 |
| **Study 3** | **4.18** | **1, 147** | **.042** |
| Symbolic-only condition | b = .01, SE = .01, 95% CI [-.01; .03] | | |
| **Symbolic-plus-material condition** | **b = .04, SE = .01, 95% CI [.02; .06]** | | |
| Moral motive |  |  |  |
| Study 1 | 1.89 | 1, 238 | .170 |
| Study 2 | 0.15 | 1, 261 | .703 |
| **Study 3** | **4.17** | **1, 147** | **.043** |
| Symbolic-only condition | b = .01, SE = .01, 95% CI [-.01; .03] | | |
| **Symbolic-plus-material condition** | **b = .-01, SE = .01, 95% CI [-.01; -.0001]** | | |
| Outgroup-focused motive |  |  |  |
| Study 1 | 0.41 | 1, 238 | .524 |
| Study 2 | 0.02 | 1, 261 | .893 |
| Study 3 | 2.60 | 1, 147 | .109 |
| Authenticity |  |  |  |
| Study 1 | 21.76 | 1, 238 | .186 |
| Study 2 | 0.15 | 1, 261 | .695 |
| Study 3 | 2.06 | 1, 147 | .153 |
| Effectiveness |  |  |  |
| Study 1 | 2.29 | 1, 238 | .132 |
| Study 2 | 0.07 | 1, 261 | .785 |
| Study 3 | 3.82 | 1, 147 | .053 |
| Legitimacy |  |  |  |
| Study 1 | 0.35 | 1, 238 | .557 |
| Study 2 | 0.01 | 1, 261 | .927 |
| Study 3 | 1.13 | 1, 147 | .290 |
| Desirability |  |  |  |
| Study 1 | 1.48 | 1, 238 | .224 |
| Study 2 | 0.40 | 1, 261 | .528 |
| Study 3 | 0.91 | 1, 147 | .341 |

Political orientation x condition

| Outcome | *F* | *df* | *p* |
| --- | --- | --- | --- |
| Self-focused motive |  |  |  |
| Study 1 | 0.03 | 1, 239 | .956 |
| Study 2 | 0.49 | 1, 261 | .484 |
| Moral motive |  |  |  |
| Study 1 | 0.01 | 1, 239 | .931 |
| Study 2 | 0.14 | 1, 261 | .705 |
| Outgroup-focused motive |  |  |  |
| Study 1 | 0.09 | 1, 239 | .766 |
| Study 2 | 1.03 | 1, 261 | .311 |
| Authenticity |  |  |  |
| Study 1 | 0.01 | 1, 239 | .915 |
| Study 2 | 0.01 | 1, 261 | .944 |
| Effectiveness |  |  |  |
| Study 1 | 0.00 | 1, 239 | .995 |
| Study 2 | 0.51 | 1, 261 | .478 |
| Legitimacy |  |  |  |
| Study 1 | 0.75 | 1, 239 | .387 |
| Study 2 | 0.003 | 1, 261 | .956 |
| Desirability |  |  |  |
| Study 1 | 0.52 | 1, 239 | .472 |
| Study 2 | 0.06 | 1, 261 | .804 |

Identity centrality x condition

| Outcome | *F* | *df* | *p* |
| --- | --- | --- | --- |
| Self-focused motive |  |  |  |
| Study 1 | 0.19 | 1, 239 | .666 |
| Study 2 | 0.70 | 1, 261 | .403 |
| Moral motive |  |  |  |
| Study 1 | 3.33 | 1, 239 | .069 |
| Study 2 | 0.002 | 1, 261 | .960 |
| Outgroup-focused motive |  |  |  |
| Study 1 | 0.20 | 1, 239 | .658 |
| Study 2 | 1.26 | 1, 261 | .262 |
| Authenticity |  |  |  |
| Study 1 | 1.72 | 1, 239 | .191 |
| Study 2 | 0.21 | 1, 261 | .647 |
| Effectiveness |  |  |  |
| Study 1 | 1.39 | 1, 239 | .240 |
| Study 2 | 0.17 | 1, 261 | .682 |
| Legitimacy |  |  |  |
| Study 1 | 0.01 | 1, 239 | .907 |
| Study 2 | 0.01 | 1, 261 | .920 |
| Desirability |  |  |  |
| Study 1 | 2.98 | 1, 239 | .085 |
| Study 2 | 0.30 | 1, 261 | .583 |

Corporate allyship attitude x condition

| Outcome | *F* | *df* | *p* |
| --- | --- | --- | --- |
| Self-focused motive |  |  |  |
| Study 1 | 0.15 | 1, 239 | .699 |
| Study 2 | 2.10 | 1, 261 | .149 |
| Moral motive |  |  |  |
| Study 1 | 0.03 | 1, 239 | .860 |
| Study 2 | 0.03 | 1, 261 | .862 |
| Outgroup-focused motive |  |  |  |
| Study 1 | 0.14 | 1, 239 | .711 |
| Study 2 | 1.18 | 1, 261 | .279 |
| Authenticity |  |  |  |
| Study 1 | 0.14 | 1, 239 | .713 |
| Study 2 | 1.11 | 1, 261 | .294 |
| Effectiveness |  |  |  |
| Study 1 | 0.07 | 1, 239 | .788 |
| Study 2 | 1.41 | 1, 261 | .236 |
| Legitimacy |  |  |  |
| Study 1 | 0.05 | 1, 239 | .826 |
| Study 2 | 2.01 | 1, 261 | .157 |
| Desirability |  |  |  |
| Study 1 | 1.07 | 1, 239 | .301 |
| Study 2 | 1.53 | 1, 261 | .218 |

Group (gender) x condition (Study 3)

| Group | Disadvantaged (women + nonbinary) | Advantaged (men) |
| --- | --- | --- |
| Symbolic-only condition | 53 | 19 |
| Symbolic-plus-material condition | 98 | 30 |

| Outcome | *F* | *df* | *p* |
| --- | --- | --- | --- |
| Self-focused motive |  |  |  |
| **Condition** | **49.90** | **1, 196** | **<.001** |
| Group | 1.12 | 1, 196 | .294 |
| Condition x Group | 0.02 | 1, 196 | .896 |
| Moral motive |  |  |  |
| Condition | 0.61 | 1, 196 | .435 |
| Group | 0.04 | 1, 196 | .845 |
| Condition x Group | 0.02 | 1, 196 | .902 |
| Outgroup-focused motive |  |  |  |
| **Condition** | **29.09** | **1, 196** | **<.001** |
| Group | 0.18 | 1, 196 | .675 |
| Condition x Group | 0.04 | 1, 196 | .846 |
| Authenticity |  |  |  |
| **Condition** | **13.07** | **1, 196** | **<.001** |
| Group | 0.10 | 1, 196 | .756 |
| Condition x Group | 0.01 | 1, 196 | .937 |
| Effectiveness |  |  |  |
| **Condition** | **7.30** | **1, 196** | **.007** |
| Group | 1.30 | 1, 196 | .255 |
| Condition x Group | 0.71 | 1, 196 | .400 |
| Legitimacy |  |  |  |
| **Condition** | **13.06** | **1, 196** | **<.001** |
| Group | 0.29 | 1, 196 | .590 |
| Condition x Group | 0.67 | 1, 196 | .413 |
| Desirability |  |  |  |
| Condition | 2.64 | **1, 196** | .106 |
| Group | 0.17 | 1, 196 | .678 |
| Condition x Group | 1.04 | 1, 196 | .309 |
