# Supplementary material for: Put your money where your merch is? Disadvantaged group members' evaluations of symbolic corporate allyship with and without a material component
Source: Br J Soc Psychol. 2026 Oct 25;65(4):e70149. doi: 10.1111/bjso.70149 (PMC13696842; doi:10.1111/bjso.70149)
Supplement: Supplementary file 5 [file BJSO-65-0-s006.docx]

SOM Table A

*Cell means and standard deviations as a function of allyship type, with mean difference tests – without attention check exclusions*

|  | Study 1 | |  |  | Study 2 | |  |  | Study 3 | |  |  |
| --- | --- | --- | --- | --- | --- | --- | --- | --- | --- | --- | --- | --- |
| Outcome | M (SD) | |  |  | M (SD) | |  |  | M (SD) | |  |  |
|  | sym.  (139) | mat.  (159) | *F*  (1, 296) | p | sym.  (278) | mat.  (232) | F  (1, 508) | p | sym.  (102) | mat.  (94) | F  (1, 194) | p |
| Self-focused motive | **5.81 (0.98)** | **5.05 (1.10)** | **38.47** | **<.001** | 4.76  (1.34) | 4.75 (1.39) | 0.01 | .936 | **5.54 (1.09)** | **4.27**  **(1.38)** | **51.28** | **<.001** |
| Morality motive | **3.91 (1.20)** | **4.39 (1.10)** | **12.76** | **<.001** | 5.24  (1.37) | 5.41  (1.26) | 2.06 | .152 | 4.38 (1.15) | 4.46 (1.23) | 0.25 | .616 |
| Outgroup-focused motive | **2.73 (1.06)** | **3.86 (1.15)** | **76.93** | **<.001** | **4.77 (1.34)** | **5.07**  **(1.25)** | **6.56** | **.011** | **3.17**  **(1.28)** | **4.21 (1.29)** | **32.35** | **<.001** |
| Authenticity | **2.80**  **(1.11)** | **3.64**  **(1.17)** | **39.80** | **<.001** | 4.68  (1.35) | 4.64  (1.36) | 0.08 | .778 | **3.07**  **(1.12)** | **3.77**  **(1.32)** | **16.33** | **<.001** |
| Effectiveness | **3.42 (1.18)** | **3.75 (1.21)** | **5.81** | **.017** | 5.16  (1.36) | 5.18  (1.32) | 0.05 | .831 | **3.61**  **(1.11)** | **4.15**  **(1.31)** | **9.73** | **.002** |
| Legitimacy | **3.85 (1.16)** | **4.23 (1.20)** | **7.76** | **.006** | 5.25 (1.32) | 5.08 (1.32) | 2.04 | .154 | **4.52**  **(1.01)** | **4.93**  **(1.20)** | **6.77** | **.010** |
| Desirability | **4.31**  **(1.27)** | **4.79**  **(1.39)** | **9.70** | **.002** | 5.62  (1.38) | 5.64  (1.30) | 0.02 | .890 | 4.97  (1.25) | 5.29  (1.16) | 3.43 | .066 |
